# Aspartimide Modification in RiPP Natural Products

**DOI:** 10.1021/acs.biochem.6c00055

**Published:** 2026-03-06

**Authors:** Angela Zhu, A. James Link

**Affiliations:** † Department of Chemical and Biological Engineering, 6740 Princeton University, Princeton, New Jersey 08544, United States; ‡ Department of Chemistry, Princeton University, Princeton, New Jersey 08544, United States; § Department of Molecular Biology, Princeton University, Princeton, New Jersey 08544, United States; ∥ Omenn-Darling Bioengineering Institute, Princeton University, Princeton, New Jersey 08544, United States; ⊥ Andlinger Center for Energy and the Environment, Princeton University, Princeton, New Jersey 08544, United States

**Keywords:** RiPPs, lanthipeptide, lasso peptide, graspetide, aspartimide

## Abstract

Multiple classes of ribosomally synthesized and post-translationally modified peptides (RiPPs) are chemically modified with an enigmatic functional group, the aspartimide. This modification occurs via the action of an enzyme related to the protein repair catalyst protein isoaspartyl methyltransferase (PIMT). Contrary to canonical PIMTs which methylate isoaspartate residues within a protein, RiPP-associated PIMTs directly methylate specific Asp residues within the RiPP substrate, resulting in the formation of an aspartimide. The biochemical details of aspartimidylation in three RiPP classes, lanthipeptides, lasso peptides, and graspetides, are described herein. The discovery of a new class of RiPPs, the imiditides or type I pamtides, with aspartimide as the class-defining post-translational modification, is also described. Finally, knowledge gaps as well as suggestions for future research are discussed.

## Graphical abstract

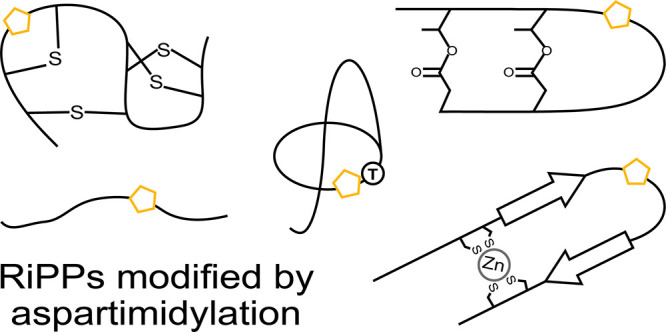

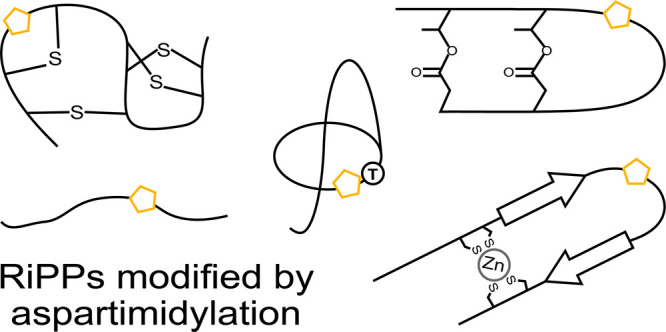

## Introduction

The aspartimide, or aspartyl succinimide (Figure 1), is an electrophilic functional group that is often associated with nuisance products in solid-phase peptide synthesis,^1–4^ protein synthesis by native chemical ligation,^5^ and in protein product formulation.^6^ While some other succinimides appear as stable functional groups in natural products,^7,8^ aspartimides found in peptides and proteins are often an intermediate, due to the electrophilic nature of the group and its ease in being opened to either aspartate (Asp) or isoaspartate (isoAsp) by attack with water. Aspartimides are well-known as intermediates in spontaneous protein aging occurring from deamidation at asparagine (Asn) residues or dehydration at Asp residues (Figure 1).^9^ Aspartimides can also be formed upon glycosylation at Asp,^10^ and a C-terminal aspartimide is formed in the course of intein-mediated protein splicing.^11,12^ The hydrolysis of an aspartimide into isoAsp introduces an extra methylene group into the peptide backbone and thus may result in protein aggregation or a loss of protein function. For example, isomerization to isoAsp in the long-lived eye lens proteins α- and β-crystallin is correlated with the formation of cataracts.^13–15^ Nature has evolved an enzyme, protein isoaspartyl methyltransferase (PIMT), as a protein repair catalyst to undo the formation of isoAsp residues within proteins.^16–18^ PIMT recognizes isoAsp (and not Asp) residues within a protein, methylating them and driving them back to the aspartimide (Figure 1). With repeated cycles of methylation, dehydration, and hydrolysis, the PIMT is able to return the protein to a conventional backbone.

**Figure 1:**
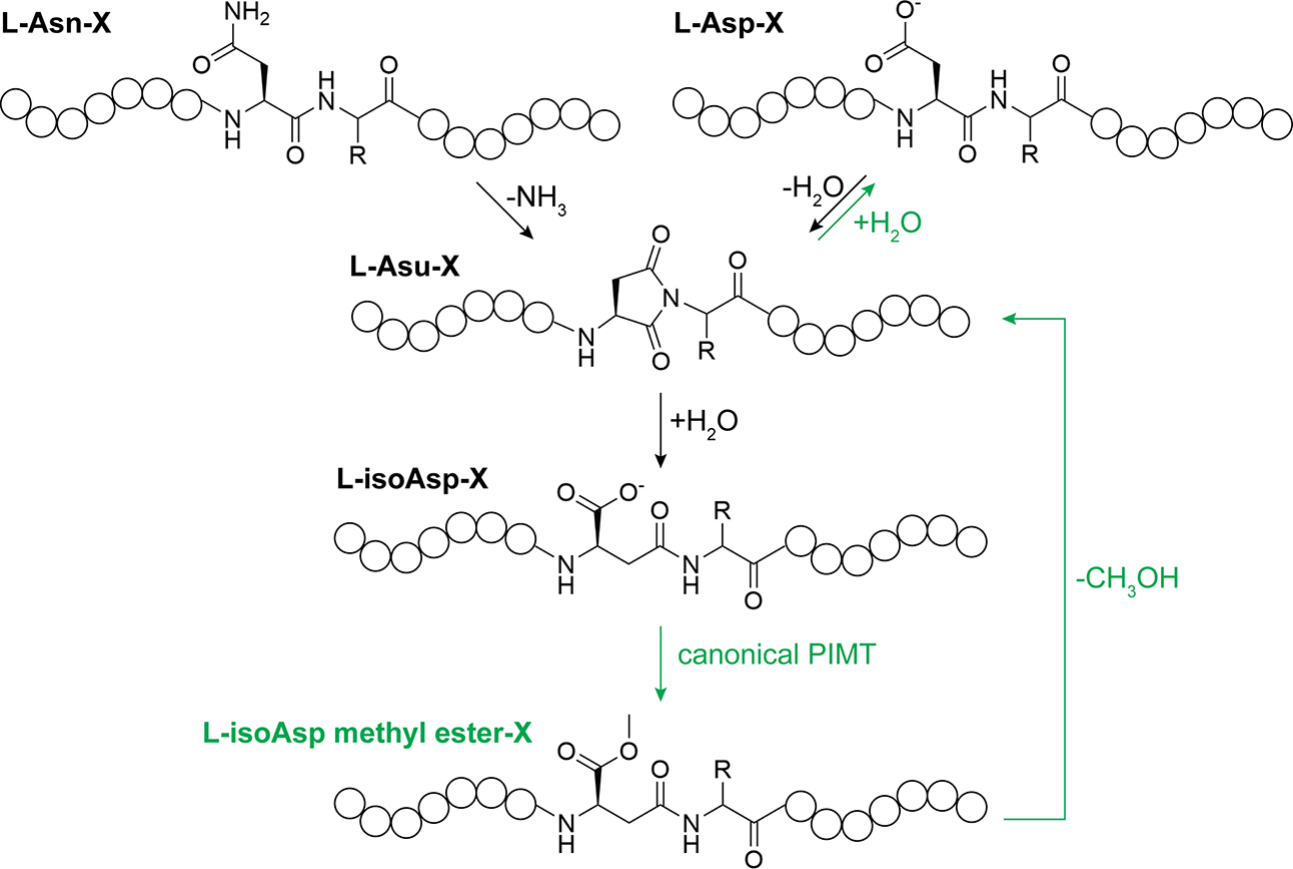
Canonical PIMTs methylate l-isoaspartate (isoAsp) residues that form after spontaneous deamidation of Asn or dehydration of Asp. These methylated residues spontaneously convert into aspartimide (i.e., aspartyl succinimide, or Asu) residues, which can hydrolyze back into Asp or isoAsp. In protein repair, repeated cycles of PIMT methylation and aspartimide hydrolysis (pathway denoted with green labels) can drive isoAsp residues into Asp residues.

While the introduction of either aspartimide or isoAsp into a polypeptide is generally considered deleterious for protein function, there are examples in the literature of improved or altered function in proteins harboring these functional groups. In a glutaminase enzyme of the hyperthermophilic archaeon *Methanocaldococcus jannaschii*, a stable succinimide moiety is present in a solvent-exposed surface loop of the protein.^19^ This succinimide is not only resistant to hydrolysis, but serves to reinforce the stability of the protein, which remains folded even at 100 °C and in 8 M guanidinium chloride.^20^ In another example, a glycosyl hydrolase found in a different hyperthermophile, *Thermus aquaticus*, generates a stable succinimide in the polysaccharide binding pocket of the enzyme.^21^ On the opposite end of the spectrum, a particularly short-lived aspartimide intermediate occurs in the enzyme MurA on the way to generating an isoAsp residue within a β-hairpin in the protein.^22^ This modification stabilizes MurA, an essential enzyme for cell wall biosynthesis, and prevents it from aggregating. Finally, aspartimides that are generated at the C-terminus of proteins in eukaryotic cells (also referred to as C-terminal cyclic imides) have been shown to bind to the ubiquitin E3 ligase substrate adapter cereblon, ultimately targeting these proteins for degradation at the proteasome.^23–26^ A recent paper shows that these aspartimides can be generated via C-terminal methylation by the housekeeping PIMT (called PCMT1 in humans).^27^

Another class of molecules in which aspartimide and isoAsp moieties have been found is the ribosomally synthesized and post-translationally modified peptides, or RiPPs.^28,29^ RiPPs are a diverse superfamily of natural products with shared biosynthetic logic; all RiPP biosyntheses start out with the synthesis of a linear precursor peptide on the ribosome. Thus far, aspartimide moieties have been found in three established classes of RiPPs: lanthipeptides,^30^ lasso peptides,^31,32^ and graspetides.^33^ Most recently, a new class of RiPPs, named imiditides^34^ or pamtides,^35^ has been discovered in which the aspartimide is the sole post-translational modification. In all cases, the aspartimide moiety is installed via the action of a homologue of the PIMT enzyme that methylates one specific Asp residue within the RiPP (Figure 2). Here we will discuss each of the different aspartimidylated RiPP classes, focusing on the structure and biosynthesis of these natural products.

**Figure 2:**
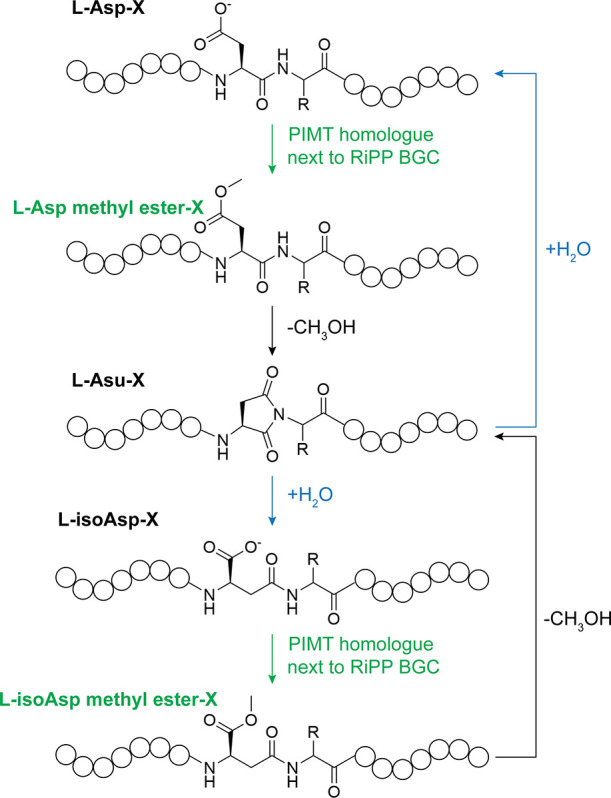
PIMT homologues adjacent to RiPP BGCs methylate a l-Asp residue in the precursor peptide. Some of these PIMT homologues can also methylate the l-isoAsp residue that appears after aspartimide hydrolysis. Both methylated species can convert into aspartimide. Action of the PIMT homologue is marked in green, and hydrolysis pathway is marked in blue.

### Nomenclature

Throughout this review we will refer to all RiPPs and their intermediates by their published names. Modifying enzymes coexpressed with the precursor are denoted either within parentheses or with superscripts.^35–37^ Modified precursors are sometimes indicated by a “m” in front of the precursor peptide name.^37,38^ Additionally, our group refers to modified core peptides that lack aspartimides with a “pre-” prefix.^39,40^ As aspartimides have been observed as an auxiliary modification in multiple classes of RiPPs, we propose adding “imiditide” to indicate RiPPs containing potential aspartimide formation, e.g., lanthimiditides for lanthipeptides, lassimiditides for lasso peptides, and graspimiditides for graspetides.^41^

### Lanthimiditides

The presence of PIMT homologues in RiPP biosynthetic gene clusters (BGCs) was first investigated in lanthipeptides. Lanthipeptides are among the largest and most well-studied classes of RiPPs. Lanthipeptides carry thioether bridges that often result in multicyclic peptides (Figure 3).^30^ In 2010, Hill and colleagues discovered that many class I lanthipeptide BGCs in *Frankia* and *Streptomyces* contained adjacent *O*-methyltransferases,^42^ and in 2015, van der Donk and colleagues confirmed the frequent presence of PIMT homologues in lanthipeptide BGCs from Actinomycetota.^43^ These authors noted the conservation of an aspartate (Asp) residue in the cores of these precursor peptides, with the conserved Asp typically followed by glycine, although *n* + 1 residues of threonine, asparagine, and aspartate were observed as well.^43^ It appeared that the methyltransferases did not coevolve with the lanthipeptide LanC biosynthetic enzymes despite their proximity.^43^

**Figure 3:**
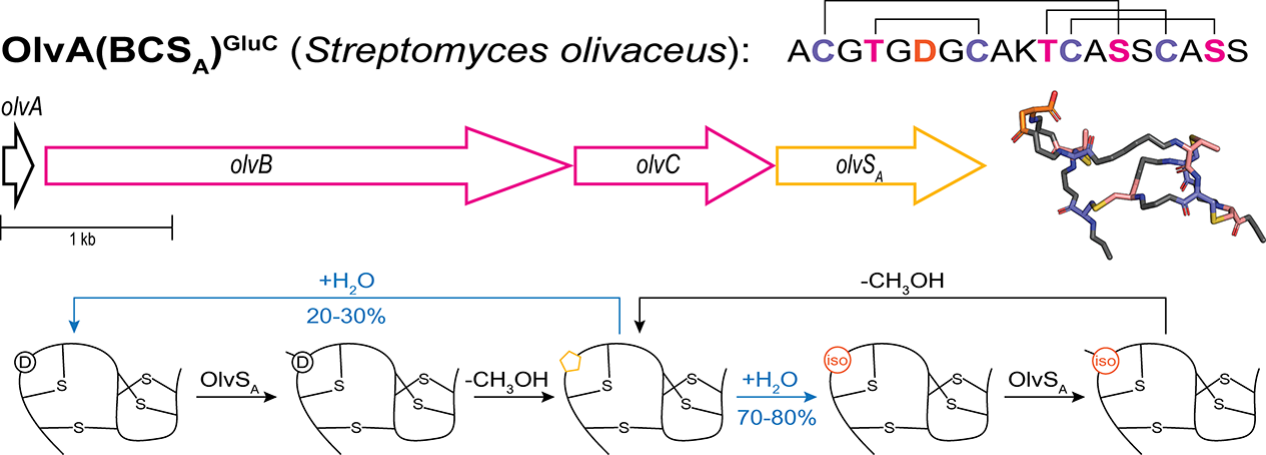
Lanthimiditide OlvA­(BCS_A_)^GluC^ goes through cycles of methylation, aspartimidylation, and regioselective hydrolysis into isoAsp. Top: biosynthetic gene cluster for OlvA­(BCS_A_) and the NMR structure of OlvA­(BCS_A_)^GluC^ containing isoAsp (PDB: 6PQF), with the isoAsp residue in orange. Bottom left: the methyltransferase OlvS_A_ can methylate both Asp and isoAsp.

Acedo et al. characterized one of these BGCs from *Streptomyces olivaceus* NRRL B-3009 with the *O*-methyltransferase OlvS_A_ (Figure 3).^36^ OlvS_A_ only acts after the dehydratase OlvB and cyclase OlvC, modifying the cyclized peptide substrate OlvA­(BC) but not unmodified OlvA or solely dehydrated OlvA­(B). Modification of OlvA­(BC) by OlvS_A_ results in methylation, followed by spontaneous aspartimide formation, which then led to hydrolysis of the aspartimide into a mixture of 70–80% isoaspartate (isoAsp) and 20–30% Asp (Figure 3).^36^ This regioselectivity in aspartimide hydrolysis is similar to what is seen for aspartimides in model peptides and aspartimides formed by canonical PIMTs.^9,44^

At room temperature in pH 7 buffer, in vitro methylation of OlvA­(BC) occurs within 15 min and finishes around 2 h.^36^ Around half of the methylated species gets converted into aspartimide by 6 h, and by 24 h, the methylated species is completely aspartimidylated with aspartimide hydrolysis starting to occur. At 37 °C, the 1:1 methylated/aspartimidylated mixture is completely hydrolyzed by 24 h. OlvS_A_ does not require leader peptide recognition, but methylation of the GluC-digested core peptide OlvA­(BC)^GluC^ is slower than that of full-length OlvA­(BC) and takes around 2 h to appear.^36^ OlvS_A_ is also able to methylate the isoAsp-containing hydrolysis product OlvA­(BCS_A_) and isoAsp-containing OlvA­(BCS_A_)^GluC^ at faster rates than their Asp-containing counterparts. The isoAsp residue in the core peptide OlvA­(BCS_A_)^GluC^ is in an N-terminal solvent-exposed loop formed by a MeLan ring (Figure 3).^36^

Regarding nomenclature, the PIMT homologues associated with lanthipeptide BGCs are called “LanS_A_,” so as not to be confused with a separate family of methyltransferases LanS_B_ that methylate the C-terminal carboxyl group or the *N*-methyltransferase family LanS_C_.^36,45,46^ In 2020, PIMT homologues were determined to be the most frequently occurring auxiliary enzyme in class I lanthipeptide BGCs, and 837 PIMT homologues next to lanthipeptide BGCs were identified.^36,47^ These methyltransferases can also appear fused to additional glutamyl lyase domains involved in lanthipeptide biosynthesis.^47,48^ Kim and colleagues later reported identification of 1305 lanthimiditide BGCs in Lee et al., with the conserved Asp residue in a TXDGC core motif.^35,47^

### Lassimiditides

Lasso peptides are a class of RiPPs named after their threaded structure resembling a lasso or a slipknot.^31,32^ This structure includes an isopeptide bond between the N-terminus of the peptide and an acidic side chain, generating an N-terminal ring through which the C-terminal portion of the peptide is threaded. There are currently two characterized aspartimidylated lasso peptides, or lassimiditides, cellulonodin-2 from *Thermobifida cellulosilytica* and lihuanodin from *Lihuaxuella thermophila* (Figure 4).^39^ These two peptides share a conserved DTAD motif at core positions 6–9. Genome mining revealed 58 other lassimiditides reported by Cao et al. in 2023; seven of these other peptides contain Ser at position 7.^49^ The Asp at position 6 is modified by the PIMT homologue, and the Asp at position 9 forms the lasso peptide ring (Figure 4).^39^ Regarding nomenclature, our group refers to the aspartimidylated lasso peptides as cellulonodin-2 and lihuanodin, and to the modified core peptides without the aspartimides (i.e., just the lassoed species) as pre-cellulonodin-2 and pre-lihuanodin.^39^

**Figure 4:**
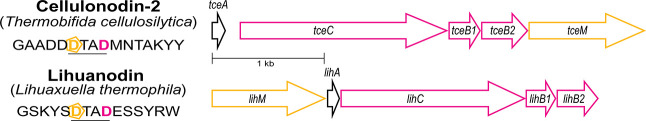
Two lassimiditides have been heterologously expressed. Their biosynthetic gene clusters and core peptide sequences are shown. The aspartimidylated Asp residue is marked within the core peptide with a yellow pentagon, and the ring-forming Asp residue is shown in pink. The conserved DTAD motif is underlined.

Heterologous expression of both cellulonodin-2 and lihuanodin in *Escherichia coli* produces the aspartimidylated species, and the aspartimide moieties remain stable in water.^39^ Racemization of the aspartimide is not observed in lihuanodin.^49^ The aspartimides hydrolyze in 50 mM Tris–HCl buffer at pHs 6–9, completely hydrolyzing in basic conditions over 21 h at room temperature.^39^ The relative stability of these aspartimides can be explained kinetically; the methylation rate constant for lihuanodin (25.6 min^–1^) is around twice the order of magnitude of its aspartimidylation rate constant, which is itself around twice the order of magnitude of its aspartimide hydrolysis rate constant.^49^ Notably, lassimiditides appear to be the only RiPP class in which the aspartimide hydrolyzes regioselectively to Asp and not isoAsp.^34–37,39–41,49,50^ This regioselectivity is uniquely desirable because hydrolysis into isoAsp increases the size of the lasso peptide ring by 1 atom and leads to unthreading of the lasso peptide.^49^ The conserved threonine (Thr) after the aspartimide ensures hydrolysis into Asp, allowing for repeated methylation and aspartimidylation of pre-lihuanodin (Figure 5).^39,49^ In contrast, changing lihuanodin’s conserved threonine into leucine biases the aspartimide hydrolysis toward isoAsp, and its methyltransferase LihM cannot act on the unthreaded isoAsp-containing peptide (Figure 5).^49^

**Figure 5:**
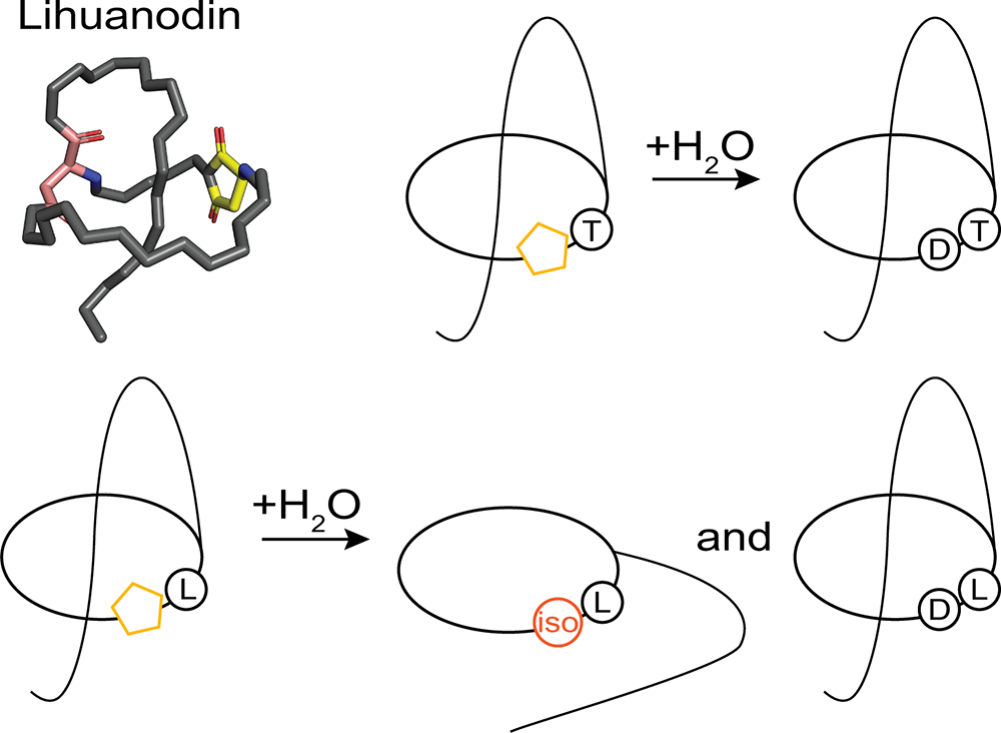
Thr7 in lihuanodin ensures regioselective hydrolysis of the aspartimide into Asp. This preserves the threaded nature of the lasso peptide and allows for potential repeated cycles of modification. Substituting Thr7 with leucine allows for hydrolysis into isoAsp and unthreading of the lasso peptide. The NMR structure of lihuanodin is shown (PDB: 7LCW), with the aspartimide in yellow and the ring-forming Asp residue in pink.

The entire lasso structure is required for modification, as the cellulonodin-2 methyltransferase TceM does not modify its associated linear precursor peptide, linear core peptide, or isopeptide-bonded ring alone.^39^ Lassimiditide methyltransferases also share a conserved WXXXGXP motif in the C-terminal domain that plays a role in modification; substitution of W in this motif with alanine in TceM abrogates aspartimide formation, though the same is not true for LihM.^39^ Additionally, TceM and LihM can act on each other’s substrates, suggesting that the shared WXXXGXP motif is important for substrate recognition.^39^

Kim and colleagues later identified 67 lassimiditide BGCs, with group 1 precursors containing the modified Asp in the conserved D­(T/S)­AD ring motif (core position 6–9) such as in cellulonodin-2 and lihuanodin, while group 2 precursors are predicted to contain the modified Asp in the loop instead.^35^ A potential lassimiditide belonging to the latter group was recently noted in the genome of *Actinoalloteichus caeruleus* LHW52806.^51^

### Graspimiditides

Graspetides are a class of RiPPs named after the ATP-grasp enzyme that modifies the class.^33^ These ATP-grasp enzymes catalyze the formation of ester and amide linkages between pairs of side chains. Multiple genome mining studies have reported the presence of *O*-methyltransferases near graspetide BGCs, as early as 2009 by Aravind and colleagues.^35,41,52–54^ Koonin and colleagues found that PIMT homologues are one of the most common proteins associated with ATP-grasp enzymes, along with double glycine peptidases and ABC transporters.^54^ Mitchell and colleagues classified these BGCs as group 13 graspetides (out of 24 groups), further categorizing the precursors into five main subgroups based on leader peptide sequence motifs.^53^ Of the 1326 group 13 graspetide BGCs they identified, 99% were from Actinomycetota, with the remaining few from Chloroflexi followed by Acidobacteria.^53^ Our group searched for putative graspimiditides using the PIMT homologue as the bioinformatic hook, identifying 962 BGCs that sort into eight clusters based on core peptide sequence motifs.^41^ Kim and colleagues have also used the PIMT homologue to bioinformatically identify 1432 graspimiditide BGCs.^35^

Four graspimiditides have been characterized: fuscimiditide from *Thermobifida fusca*,^40^ amycolimiditide from *Amycolatopsis cihanbeyliensis*,^37^ albusimiditide from *Streptomyces albus* J1074 (renamed to *Streptomyces albidoflavus* J1074),^41^ and SsfA­(BM) from *Streptomyces* sp. F-3 (Figure 6).^35^ These four peptides have stem-loop macrocyclic structures made of ω-ester cross-links between Thr and Asp residues, with the Asp methylation site present in the loop.^35,37,40,41^ The number of ester cross-links varies from two to five; the fifth linkage in SsfA­(BM)_63–97_ is postulated to be a side-chain-to-C-terminus linkage.^35^ Although these currently characterized graspimiditides all have glycine following the methylated Asp (Figure 6), analysis of core peptide sequence motifs suggests that serine, arginine, threonine, asparagine, lysine, methionine, and histidine are also possible *n* + 1 residues.^41^

**Figure 6:**
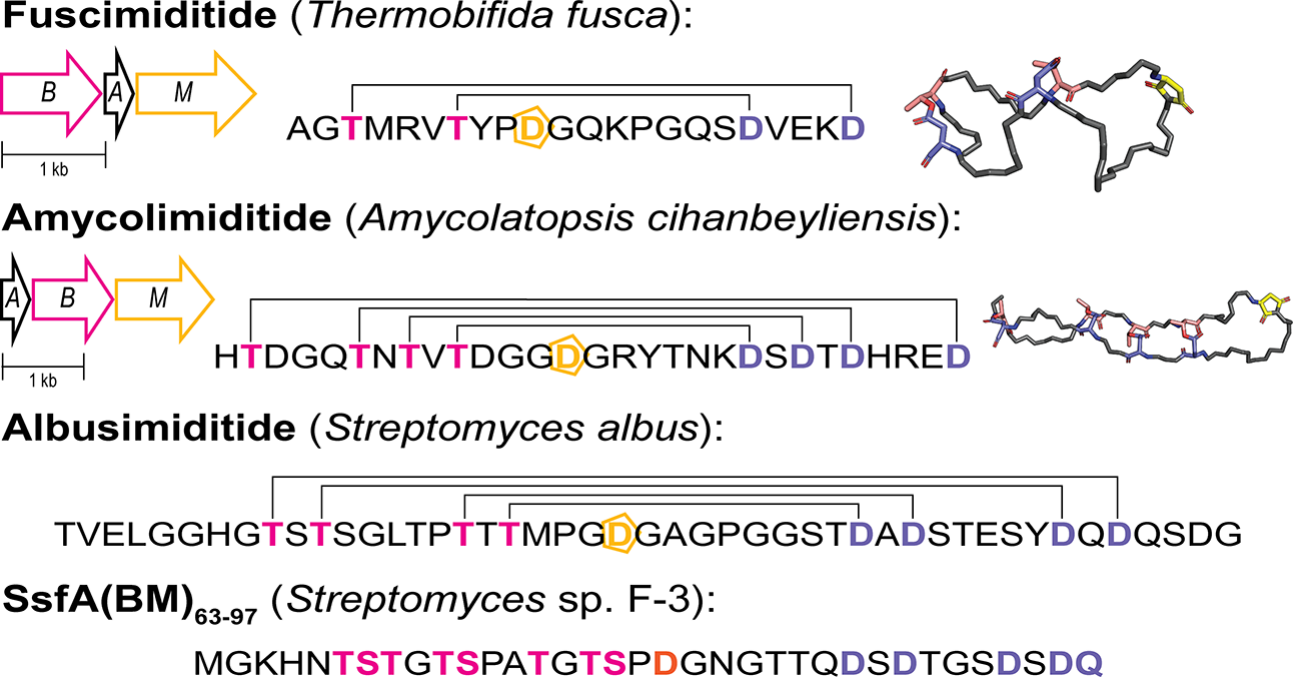
Four graspimiditides have been heterologously expressed. Albusimiditide and SsfA­(BM)_63–97_ share the same biosynthetic gene cluster structure as amycolimiditide. The putative core sequences, obtained from trypsin or GluC digests, are shown, along with any determined ester linkages. SsfA­(BM)_63–97_ is postulated to have five cross-links and was purified as a mixture containing isoAsp and Asp at the position of the orange D. The other three peptides were purified in the aspartimidylated form (marked with yellow pentagons). NMR structures of fuscimiditide (PDB: 7LIF) and amycolimiditide (PDB: 8DYM) are shown with the aspartimides in yellow and the Thr and Asp residues forming the ester cross-links in pink and purple.

Similar to what is seen for lanthipeptides and lasso peptides, the graspetide leader peptides are not necessary for methylation and aspartimidylation to occur.^35,37,40^ In contrast to what is seen for lanthipeptide OlvA­(BC)^GluC^, in vitro aspartimidylation of pre-amycolimiditide (i.e., trypsin-digested mAmdA^B^) occurs at a similar rate to that of undigested mAmdA^B^.^36,37^ Aspartimide hydrolysis from fuscimiditide goes to 95% isoAsp and 5% Asp, while the ratio from amycolimiditide is the more typical 7:3 (Figure 7).^37,40^ Both the fuscimiditide and amycolimiditide methyltransferases can modify isoAsp-containing peptide, although aspartimide formation in isoAsp-containing pre-amycolimiditide is not significantly faster than its Asp counterpart as it is for isoAsp-containing OlvA­(BCS_A_)^Glu^.^36,37,40^

**Figure 7:**
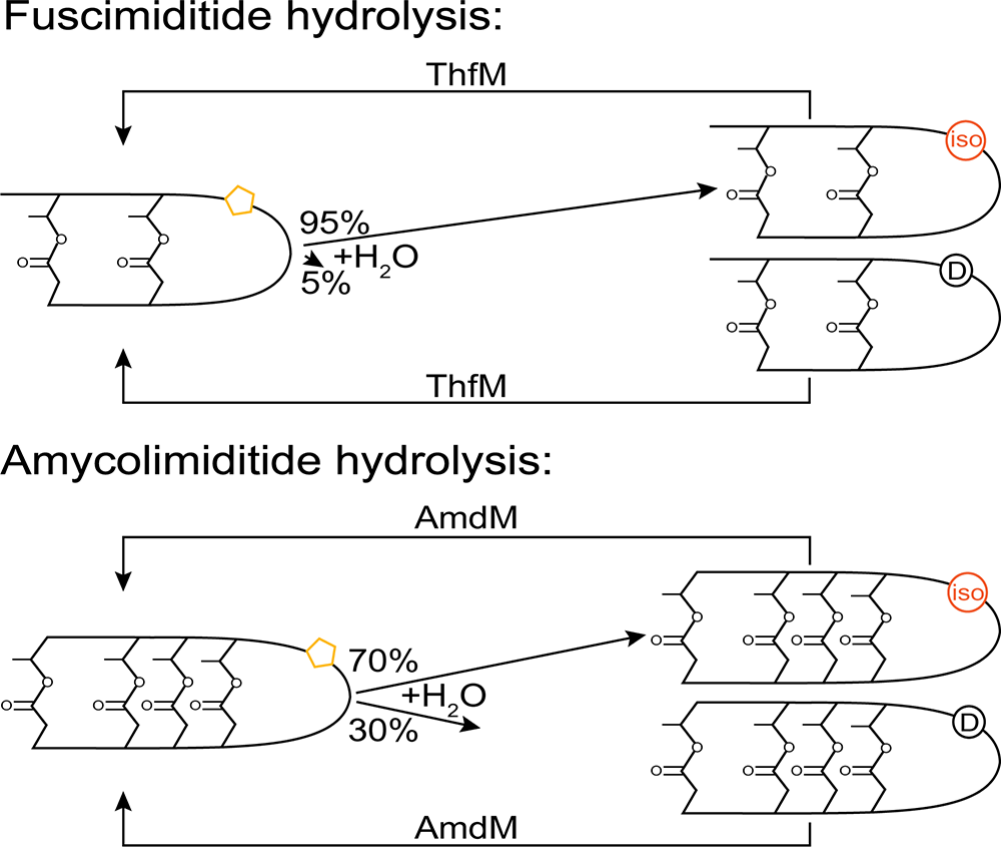
Aspartimide hydrolysis ratio in graspimiditides is preferential toward isoAsp. The aspartimide in fuscimiditide hydrolyzes predominantly into isoAsp, while the aspartimide in amycolimiditide hydrolyzes to ∼70% isoAsp. The associated methyltransferases ThfM and AmdM can also methylate the isoAsp-containing substrates.

The associated methyltransferases do not modify the linear precursor peptides.^35,37,40^ It was demonstrated for amycolimiditide variants that formation of the innermost ω-ester cross-link is still insufficient for aspartimidylation to occur; at least two of its four cross-links are required.^37^ The methylation rate of pre-amycolimiditide variants can decrease around 10-fold upon removal of a single cross-link,^37^ and removal of cross-links in SsfA­(B)_63–97_ variants has a deleterious effect on its modification as well.^35^ Shifting the AmdA aspartimidylation site by one or two residues was also generally not tolerated *in cellulo*, overall suggesting methyltransferase recognition of highly specific graspetide structures.^37^

### Imiditides

PIMT homologues have been recently used as a starting point for genome mining, revealing a new class of RiPPs that is referred to as either imiditides^34^ or pamtides.^35^ The name of “pamtide,” proposed by Kim and colleagues, refers to the description of the RiPP-modifying enzyme as “protein l-aspartyl methyltransferase (PAMT),” since unlike canonical PIMTs, these enzymes can modify Asp in addition to isoAsp.^35^ While the previously described methyltransferases all act on already modified cyclic substrates (Figure 8), imiditide-associated methyltransferases can act on otherwise unmodified linear peptide substrates. Imiditide BGCs consist of the precursor peptide (often unannotated in genome sequences) and the PIMT homologue (i.e., the PAMT) (Figure 9).^34,35^

**Figure 8:**
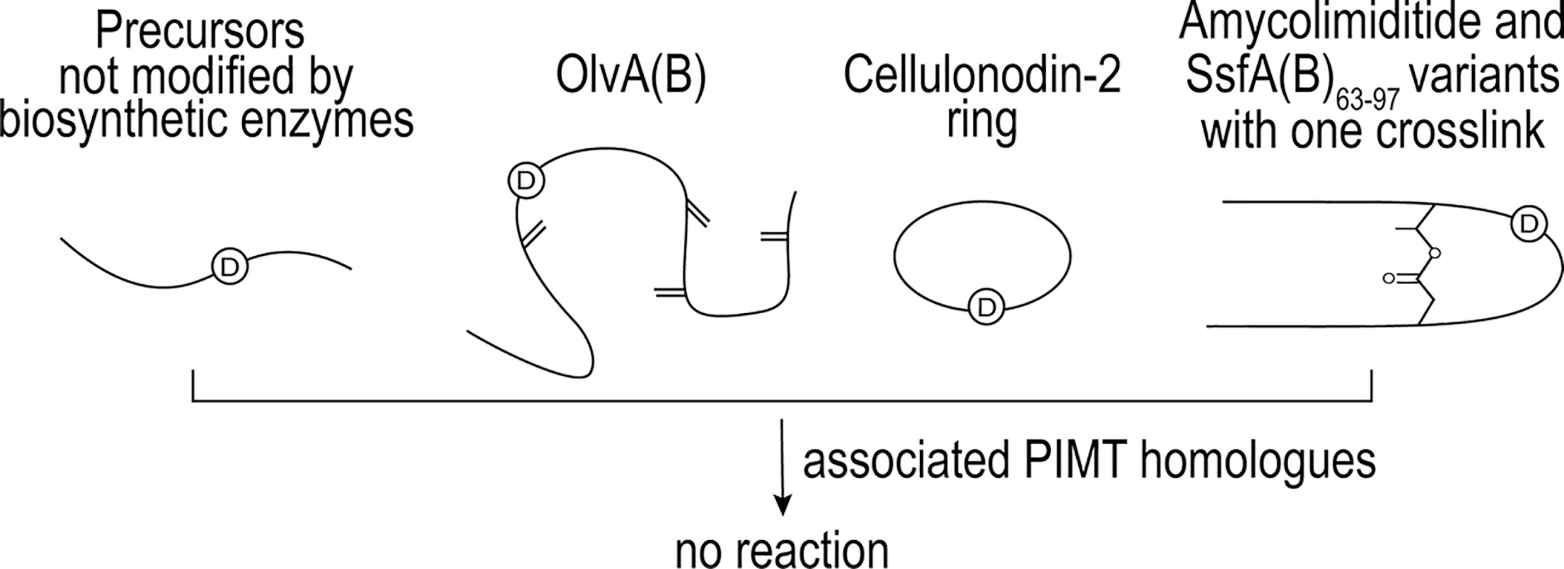
Aspartimidylation can require specific cyclic substrates. PIMT homologues associated with lanthipeptide, lasso peptide, and graspetide biosynthetic gene clusters do not modify the partially formed substrates shown here.

**Figure 9:**
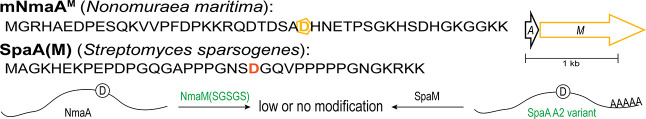
Imiditides/type I pamtides are presumably linear peptides modified by PIMT homologues. Precursor peptide sequences and a representative biosynthetic gene cluster are shown. mNmaA^M^ was purified containing partial aspartimidylation (yellow pentagon), while SpaA­(M) was purified in the hydrolyzed form (orange D). Substrate recognition is likely mediated by charge–charge interactions, as substituting charged residues in the precursor peptide or methyltransferase significantly reduces modification.

Imiditide precursor sequences are rich in glycine, proline, aspartate, and lysine (Figure 9).^34,35^ They are most frequently found in *Streptomyces* and *Actinomadura*.^34,35^ Additionally, a subgroup of these precursors contains a conserved tetracysteine motif like that of DnaJ, and is thus referred to as both cysimiditides and type II pamtides (those without the cysteine motifs are called type I pamtides).^35,50^ Cysimiditides/type II pamtides are most frequently found in *Nocardiopsis* and contain a conserved Asp residue between the two CXXCXGXG motifs (Figure 10).^35,50^ Kim and colleagues have identified a total of 1414 type I pamtides and 125 type II pamtides.^35^

**Figure 10:**
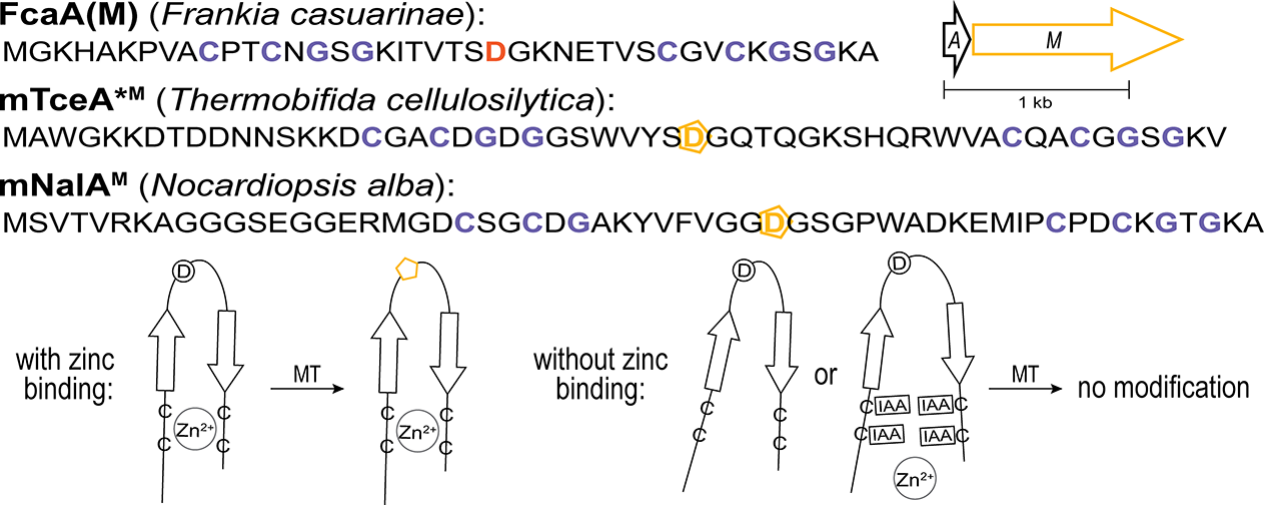
Cysimiditides/type II pamtides contain two conserved CXXCXGXG motifs that bind zinc. Precursor peptide sequences (with cysteine motifs in purple) and a representative biosynthetic gene cluster are shown. FcaA­(M) was purified containing isoAsp (at the orange D), while mTceA*^M^ and mNalA^M^ were purified containing partial aspartimidylation (yellow pentagon). The zinc binding at the cysteine motifs is important for aspartimidylation; modification does not occur if zinc is absent or if the cysteines have been alkylated with iodoacetamide (IAA).

Imiditides from *Nonomuraea maritima* and *Streptomyces sparsogenes* have been heterologously expressed in *E. coli* (Figure 9).^34,35^ After 40 h of expression, masses corresponding to aspartimidylated, unmodified, and methylated species of the *N. maritima* imiditide mNmaA^M^ can be observed in a ∼0.5:0.3:0.2 ratio.^34^ In contrast, lassimiditides and graspimiditides can demonstrate more complete conversion to the aspartimidylated species after heterologous expression.^37,39,40^ Modification of the *S. sparsogenes* pamtide SpaA­(M) was also observed in vitro, with methylated and aspartimidylated species appearing within half an hour at 25 °C and complete hydrolysis by 24 h.^35^

An uncommon histidine residue follows the modified Asp in NmaA; changing this *n* + 1 residue to threonine or glycine increases or decreases the accumulation of methylated species, respectively, while leading to a lower amount of aspartimidylated species for both.^34^ Additionally, it is possible to remove the first 20 residues of the NmaA leader peptide and still see aspartimidylation, albeit at lower levels once 15 residues have been removed.^34^ Removing the last 5 residues of NmaA did not significantly affect modification, but removing the last 10 residues did.^34^ Similarly, substituting the last 5 residues in SpaA with Ala abolished modification (Figure 9).^35^ The interaction between the imiditide precursor and its methyltransferase is believed to be mediated by charge–charge interactions.^34,35^ Substituting a negatively charged loop (DEDGD) in the C-terminal region of NmaM with a flexible linker sequence (SGSGS) also greatly decreased modification (Figure 9).^34^

In contrast to the imiditide methyltransferases which recognize linear peptide sequences, cysimiditide methyltransferases are believed to recognize hairpin-like precursor structures facilitated by zinc binding to the tetracysteine motif (Figure 10).^35,50^ Cysimiditides/type II pamtides from *Frankia casuarinae*, *T. cellulosilytica*, and *Nocardiopsis alba* have been heterologously expressed in *E. coli* (Figure 10).^35,50^ Zinc binding to the tetracysteine motif is essential; when either zinc or the cysteines are not present, no aspartimidylation is observed.^35,50^ FcaA­(M) from *F. casuarinae* was shown to convert into a majority isoAsp-containing species upon aspartimide hydrolysis.^35^ For the other two cysimiditides mTceA*^M^ and mNalA^M^, masses corresponding to aspartimidylated species (major product) and unmodified species are observed after expression, but not the methylated species.^50^ The aspartimide hydrolysis ratio for mTceA*^M^ is ∼7:3 isoAsp/Asp.^50^ Considerable hydrolysis of the aspartimides in cysimiditides can occur within 4–6 h in Tris–HCl buffer (pH 7.4–8) at room temperature.^35,50^

### RiPP-Modifying PIMT Homologues

One important consideration for the bacteria that harbor aspartimide-containing RiPPs is the substrate specificity of the PIMT responsible for aspartimidylation. Since RiPP-associated PIMTs methylate Asp residues rather than isoAsp residues, a promiscuous PIMT that methylated Asp residues throughout the proteome could be catastrophic, potentially disrupting the structures of multiple proteins. As we have discussed above, RiPP-associated PIMTs are highly specific. In the case of lanthimiditides, lassimiditides, graspimiditides, and cysimiditides, the PIMTs recognize only a single Asp residue within the core peptide sequence, and methylation only occurs on a post-translationally modified and/or folded substrate. The substrate specificity problem is addressed in imiditides/type I pamtides by electrostatic interactions between the linear peptide substrate and PIMT homologue. All RiPP-associated PIMTs carry an extra C-terminal domain relative to canonical PIMTs such as *E. coli* Pcm^55,56^ and human PIMT PCMT1 ^57^ (Figure 11). Intriguingly, lassimiditide, graspimiditide, and imiditide PIMTs all harbor a conserved WXXXGXP motif (discussed above in the lassimiditide section) near the C-terminus of this extra domain. We have proposed that this extra C-terminal domain of RiPP-associated PIMTs is involved in substrate recognition, and this has been shown directly in the case of imiditides. The PIMT from *Thermotoga maritima* also harbors a C-terminal extension containing some of the features of RiPP-associated PIMTs (Figure 11).^58,59^ For example, the *T. maritima* enzyme carries a WXXXG sequence that aligns with the WXXXGXP motif in RiPP-associated PIMTs. Ultimately, structure determination of RiPP-associated PIMTs is needed, ideally with bound substrates, to fully understand substrate recognition in these enzymes.

**Figure 11:**
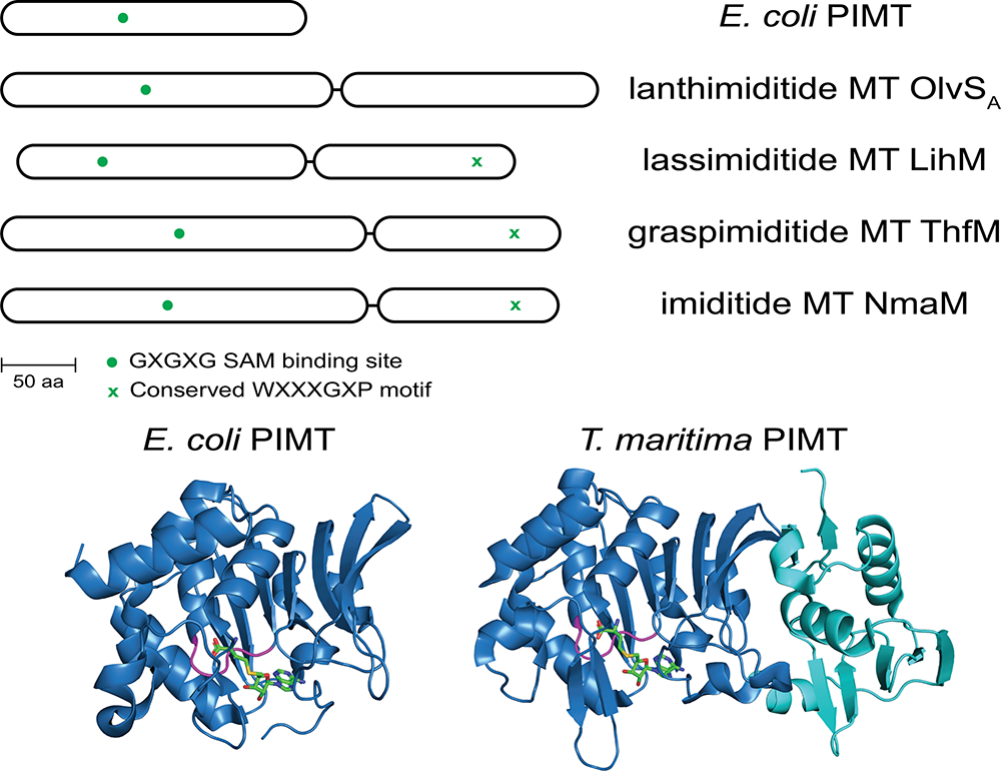
PIMT homologues next to RiPP BGCs contain an extra C-terminal domain compared to *E. coli* PIMT. Most of the C-terminal domains contain a conserved WXXXGXP motif that may contribute to substrate binding. The crystal structures of *E. coli* PIMT (PDB: 3LBF) and *Thermotoga maritima* PIMT (PDB: 1DL5) are shown with the GXGXG SAM binding site in magenta and S-adenosyl-l-homocysteine (SAH) in green sticks. The C-terminal domain of *T. maritima* PIMT (residues 215-317) is shown in cyan.

## Conclusion

In this article we have attempted to provide an overview of RiPP aspartimidylation from a biochemical perspective where great progress has been made in the past 6 years. While more biochemistry can be carried out on these systems, such as structure determination and further analysis of the RiPP-associated PIMT enzymes, the bigger challenge will be to connect aspartimidylated RiPPs to biological function. An aspartimide moiety changes the character of a peptide substantially. Relative to Asp, an aspartimide moiety is much more rigid. Conversion of Asp to aspartimide removes a negative charge from the peptide at neutral pH and also eliminates the amide proton in the n+1 position. It remains to be discovered if or how these chemical changes translate to biological function. Aspartimides could serve as electrophilic traps to covalently bind a receptor or enzyme. Since aspartimides hydrolyze, they could also be functioning as immunity factors. In this scenario, the bioactive RiPP would carry Asp or isoAsp with the aspartimidylated RiPP being inactive and protecting the native producer. Although cereblon has currently only been shown to bind to C-terminal cyclic imides, it is also tempting to speculate that aspartimidylated RiPPs could interface with cereblon and the protein degradation machinery. Perhaps these RiPPs target key proteins in eukaryotic cells and function as a molecular glue, targeting the proteins for degradation rather than just inhibition. In the case of imiditides/type I pamtides, the strong positive charges in these molecules strongly suggest that they interact with cell membranes, but the role of an aspartimide moiety in such a peptide remains unclear. Going forward, efforts should focus on connecting the chemical uniqueness of the aspartimide to bioactivities.
